# A sustained reduction in the rate of severe intraventricular hemorrhage in very low birth weight infants: a novel quality improvement project in a large perinatal-neonatal centre in Asia

**DOI:** 10.3389/fped.2025.1640964

**Published:** 2025-09-29

**Authors:** Thowfique Ibrahim, Arpan Agarwal, Abdul Alim Abdul Haium, Nirmal Kavalloor Visruthan, Maria Chona Badillo, Rowena Dela Puerta, Janlie Lizaso Banas, Sharifa Sarjono, Fareed Muhammed Bin Noorul Amin Alawdeen, Rehana Sultana, Victor Samuel Rajadurai

**Affiliations:** ^1^ Department of Neonatology, KK Women’s and Children’s Hospital, Singapore, Singapore; ^2^ DUKE-NUS Medical School, Singapore, Singapore; ^3^ YLL School of Medicine, National University Singapore, Singapore, Singapore; ^4^ LKC School of Medicine, Singapore, Singapore

**Keywords:** VLBW, ELBW, severe IVH, hypothermia, neutral head position, magnesium sulphate, indomethacin NICU neonatal intensive care unit

## Abstract

**Objective:**

Severe IVH (SIVH) stands out as a leading cause of poor neuro-developmental outcomes, including cognitive, attention and motor impairment in very low birth weight (≤1.5 kg, VLBW) infants. The study aims to reduce the rate of severe intraventricular hemorrhage (SIVH) by 50% in very low birth weight (VLBW) infants admitted to the level III C neonatal intensive care unit (NICU) in Singapore.

**Method:**

In this quality improvement (QI) study, VLBW infants admitted to NICU from 2011 to 2021 (*n* = 2215) were categorized into four periods: (a) pre-intervention 2011to 2012, (b) Intervention (2013 to 2017), (c) post-intervention (2018 to 2019), and (d) sustainment (2020 to 2021) periods, respectively. A multidisciplinary team identified key drivers for SIVH. A set of care bundles involving eight protocolized interventions was applied. Infants with SIVH were analyzed with an RC-PC-S process (Root Cause- Process Compliance - System), which includes a structured template by a Quality Assurance (QA) committee; recommendations were implemented in the unit to modify practices. Data were collected prospectively, and univariate and multivariate logistic regression analyses were conducted.

**Results:**

Of 2,215 Infants who met the study criteria, Ninety infants were excluded. Detailed data were collected from 2,125 infants (390, 1,000, 370, and 365 in the baseline, intervention, post-intervention, and sustainment periods, respectively). The mean gestational age was 28·6 and 28.8 in the intervention and post-intervention periods, respectively, and were comparable across the 4 study periods. The rate of SIVH was 5.9% in the pre-intervention period and 4.4% and 1.9% (adjusted OR 0.266, *p* = 0.006, 44/1,000 vs. 7/370) in the intervention and the post-intervention periods, respectively, representing a reduction of 57%. The rate of SIVH in the sustainment period was 2·7%. The reduced SIVH was associated with reduced mortality, adherence to process measures, and no change in balancing measures.

**Conclusion:**

A multipronged modified QI approach implementing an evidence-based SIVH prevention bundle and RC-PC-S analysis was associated with a sustained significant reduction in the rate of SIVH in VLBW infants. RC-PC-S is a potential QI tool for reducing severe IVH and other key neonatal morbidities in VLBW infants.

## Introduction

Survival without disability is the optimal result for neonatal care, especially for very low birth weight infants (≤1.5 kg, VLBW), who are at high risk for adverse outcomes due to major morbidities, notably intraventricular hemorrhage (IVH) (1, 2). Among the key morbidities in VLBW infants, SIVH stands out as a leading cause of poor neurodevelopmental outcomes. Approximately one-half of infants affected by SIVH either suffer from moderate to severe disability or die (3–7). SIVH remains a significant burden in VLBW infants, with population studies reporting incidences varying from 7·9 to 9·4% (8).

SIVH arises from a complex interplay of factors in preterm infants, ranging from fragile cerebral vasculature to hemodynamic instability and inflammatory responses. Targeted interventions employing quality improvement (QI) methodologies have emerged as a promising avenue for reducing IVH and other key neonatal morbidities. These interventions focus on evidence-based practices that can be applied at different stages of care, such as the antenatal, perinatal, and postnatal periods (9–18). However, QI strategies has often been constrained by a narrow focus on a limited number of interventions monitored over relatively short periods. This approach does not provide a robust, sustainable reduction in SIVH rates, nor does it integrate continuous process evaluation as part of routine clinical management.

Recognizing these limitations, we identified a gap in current QI methodologies. This study attempts to bridge the gap with a modified QI methodology that includes a comprehensive prevention bundle alongside a detailed case-by-case analysis to identify the root causes of SIVH, assess compliance with QI processes and standard practices, and system-associated causes [Root Cause Process compliance System (RC-PC-S) analysis]. The method also incorporates sustained monitoring, allowing for ongoing improvement without placing excessive demands on the workforce or resources. By integrating these changes, this approach seeks to achieve long-term reductions in severe IVH and promote disability-free survival in preterm infants.

A review of the rate of key morbidities in very low birth weight infants (VLBW) in our centre by analysing the data submitted to the Vermont Oxford Network database was conducted in 2012 with the overall aim of initiating QI methodologies to improve the outcome of VLBW infants in the neonatal intensive care unit of Kandang Kerbau Women's and Children's Hospital (KKH) Singapore. The KKH unit has shared the data with the Vermont Oxford network since 1998. The unit's average rate of severe IVH in the year 2011&12 was 5·9% in VLBW infants, which was in the best-performing quartile of similar units in the network in both years, but we still chose the SIVH reduction as the first QI project because of the significance of the problem in influencing the morbidity and mortality of VLBW infants as compared to other key morbidities.

The project aimed to reduce the rate of SIVH by 20% in 2 yrs and 50% within 6 years. We chose a longer duration because of the anticipated difficulty in reducing morbidity, which was already low.

## Methods

### Context and setting

The study was conducted in a, a tertiary care referral teaching hospital with a 40-bed level IIIC Neonatal Intensive Care Unit (NICU). KKH has around 12,000 deliveries per year, and an average of 200 VLBW babies are admitted to the unit every year. These babies are born to mothers cared for antenatally in the hospital's obstetric department or who have been referred to the high-risk obstetrics team from other institutions and followed up subsequently at KKH.

This project was submitted to the SingHealth Centralized Institutional Review Board, but it was considered a quality improvement project and exempt from its review.

### Conception, planning, and interventions

After a basic review of the critical outcomes for VLBW infants born in 2011 and 2012 by analysis of the data submitted to the Vermont Oxford Network, SIVH was the first of the key morbidities targeted to improve the VLBW outcome in the unit. A multidisciplinary team comprised of neonatologists, staff nurses, neonatal nurse practitioners, respiratory therapists, physiotherapists, and pharmacists was constituted. The QI committee was named “the IVH workgroup”. A comprehensive literature review was performed, and published best practices were identified (19–22)

The committee met in one to two month intervals. The following three primary drivers, estimated to represent more than 80% of the causative factors by near consensus, were identified: a)fluctuations in the cerebral blood flow, b) damage to pertinent cerebrovascular endothelial integrity (germinal matrix), and c) altered coagulation profile (Figure 2). The QI process was initially started using conventional methodology by instituting two groups of interventions, neutral head position and developmental neuroprotective care but after recognizing the complexity of the problem and the constraints inherent in traditional QI methodologies to address the clinical problem, the QI methodology had to be modified. The committee then agreed to implement the structured protocol-based interventions consecutively, With intervals varying from 6 months to 2 years. Eight interventions in total were instituted over six years (Figure 3).

In parallel to the structured interventions, all infants who developed severe IVH from January 2015 were analyzed with RC-PC-S methodology, which includes a structured checklist to identify root causes and adherence to standard practices and analysis of system issues by the committee (Supplementary Material S1, Table S3a and S3b) and a minimum attendance of 3 specialists was required for the RC-PC-S QI process meeting. The RC-PC-S root cause list for severe IVH comprised a total of nineteen terms. Six were defined based on definitions formulated by the team. Consensus recommendations were fed back to the department and managing team to modify practices and triggered structured intervention from the QI team when required. Four years after the initiation of this project, to consolidate the original intention to improve the outcome of VLBW infants, similar workgroups were set up for other key morbidities, including pneumothorax, necrotizing enterocolitis (NEC), sepsis, chronic lung disease (CLD), and retinopathy of prematurity (ROP) consecutively.

The global aim of the project was to ascertain the validity of the modified quality improvement methodology and the IVH prevention bundle instituted to reduce the incidence of severe IVH in very low birth weight (<1.5 kg, VLBW) infants and to establish its applicability in wider healthcare settings.

The SMART (specific, measurable, attainable, relevant, and time-limited) aim was to reduce the incidence of severe IVH, defined as Papile's grade 3&4 intraventricular hemorrhage, diagnosed with serial cranial ultrasound examinations, by 20% within 2 years and 50% within 6 years in very low birth weight infants admitted to our level IIIc neonatal intensive care unit.

### Interventions

An IVH prevention bundle was instituted based on the adoption of emerging potential best practices (PBP) available in contemporaneous literature reviews, along with recommendations derived from the RC-PC-S QI process. This consisted of a total of 8 interventions (Figure 3) as described below.

1. Elevated neutral head position.

The head of the bed was elevated to 15^o^. A video recording was made of a simulated neutral head position in the first 72 h of life and used by the QI team, along with other educational materials, to enhance knowledge and acceptance to all the physicians, nurses, and allied health professionals caring for these infants. Attendance of clinicians at these sessions was recorded. Compliance to the intervention was audited twice weekly for the first three months by a small team led by the RTs.

2. Developmental care

A team led by nurses educated the managing teams about developmental care (Supplementary Material S1, Table S5) summarises the key points.

3. Administration of antenatal steroid at least 48 h before delivery in ≤ 24 weeks gestational age infants at birth

In 2014, our unit policy was to offer resuscitation at 24 weeks of gestation; and less than 24 weeks in selected cases based on parental preference. We had many instances where antenatal steroid was administered at 24 weeks, and infants were born on the same day, who subsequently developed SIVH. The committee liaised with the obstetrics team and agreed to initiate antenatal steroid dose at 23 weeks and five days gestation to avoid the birth of infants with incomplete or no antenatal steroids. If the resuscitation was offered to infants less than 24 weeks, the antenatal steroid was offered when the decision to resuscitate was made. The subsequent audits revealed that policy change succeeded, and obstetricians followed the guidelines.

4. Root cause and process compliance System (RC-PC-S) analysis

Starting in 2015, an initiative was implemented that involved conducting a detailed Root Cause and Process Compliance System (RC-PC-S) analysis for all cases of severe intraventricular hemorrhage (IVH) in premature infants (Supplementary Material S1, Table S3a and S3b). The RC-PC-S approach aimed to identify both direct and upstream factors contributing to IVH, with a focus on understanding not only the known physiological risk factors but also the systemic failures or process gaps that allowed these risk factors to manifest. By scrutinizing compliance with established preventive protocols, the analysis provided insights into areas where clinical processes deviated from the standards of care, thereby contributing to severe IVH. This RC-PC-S methodology was not limited to IVH alone but was also extended to address and reduce the incidence of other key neonatal morbidities over time. The continuous evaluation and adjustment of processes based on RC-PC-S findings allowed for sustained improvements in clinical outcomes and process adherence, allowing a proactive, long-term approach to outcome improvement in the study population.

The following statements represent the recommendations RC-PC-S analysis process formulated over the six year period (Supplementary Material S1, Table S4).

5. Antenatal Magnesium Sulfate administration for Neuroprotection (23–25)

In 2015, as evidence for magnesium sulfate's role in protecting the fetal brain was evident, team members initiated a discussion with obstetricians under the umbrella of the Perinatal Society of Singapore. The discussion led to the implementation of a protocol for the administration of Magnesium Sulfate to mothers at risk of preterm delivery under 32 weeks anticipated to deliver within the subsequent 24 h (as well as for mothers with severe preeclampsia at any gestation). Compliance with the protocol was monitored to ensure its implementation.

6. Hypothermia Management Protocol (26)

From 2013 to 2015 period, hypothermia was found to be one of the commonest risk factors for SIVH in the unit. Hypothermia was defined as recording an axillary temperature ≤ 36°C in VLBW infants. The existing preventive measures in place were found to be insufficient to prevent Hypothermia during the following points: during transport from the delivery room to the NICU, during procedures with an open patient access portal, and when the neonate was undergoing a sterile procedure. The team successfully addressed the gaps with the measures described in Supplementary Material S1, Table S5. The team's effort to introduce the exothermic mattress was delayed due to regulatory issues, but it was eventually overcome after the completion of the project.

7. Implementation of PDA management protocol (27–29)

Before 2012, our PDA management policy was based on an algorithm that allowed treatment of all haemodynamically significant PDAs in premature infants, irrespective of gestational age. With this policy, we faced problems of pulmonary hemorrhage and SIVH in high-risk infants associated with delayed treatment and Cox inhibitor-associated perforations in treated infants. There was also a period during which indomethacin was not available, and Ibuprofen was the only available cox inhibitor for use.As the evidence mounted, along with a lack of consensus, that all PDAs do not need treatment, the first author advocated selective treatment policy in 2009 and the author and unit colleagues published a review on the topic (30). The ideas published in the review article influenced the individual physicians approach to PDA management, but the unit largely continued with a varied management approach to PDA treatment. Eventually, in 2016, the IVH team established a protocol based on consensus, and outcomes were published (31). The key feature of the protocol was the early selective treatment of high-risk VLBW (birth weight ≤800 g or gestation less than 27 weeks, hemodynamically significant PDA with ductal diameter >1.6 mm, and mechanical ventilation and expectant management of low-risk infants).

### 8a & 8b. Indomethacin for IVH prevention protocol

Implementation of prophylactic indomethacin protocol for IVH prevention guidelines (8a) and its subsequent modification (8b).

The eight intervention began in April 2016. After a review of the literature and audit of the local data, the neonatal group concluded that prophylactic dose indomethacin was beneficial in preventing SIVH in a select group of high-risk infants. The group defined the high risk as those born at gestational ages of <28 weeks or weights <1,000 g whose mothers had not received a full course of antenatal corticosteroid. The protocol recommended the administration of prophylactic indomethacin (0.1 mg/kg/day 24 h × 3 doses), with the first dose administered within 6 h of birth in the select high-risk group. A follow-up echocardiogram was performed 72 h after the completion of treatment. If the PDA criteria for treatment were met, high-risk infants were eligible to receive an additional course of indomethacin beyond the first week of life. In 2017, after an extensive literature review and analysis of the unit's gut perforation data, the protocol was modified to restrict the high-risk infants who meet the criteria further to those at gestational age ≤25 weeks who's mothers had had an incomplete course of steroids or none at all.

### Study population and study period

**Figure 1:**
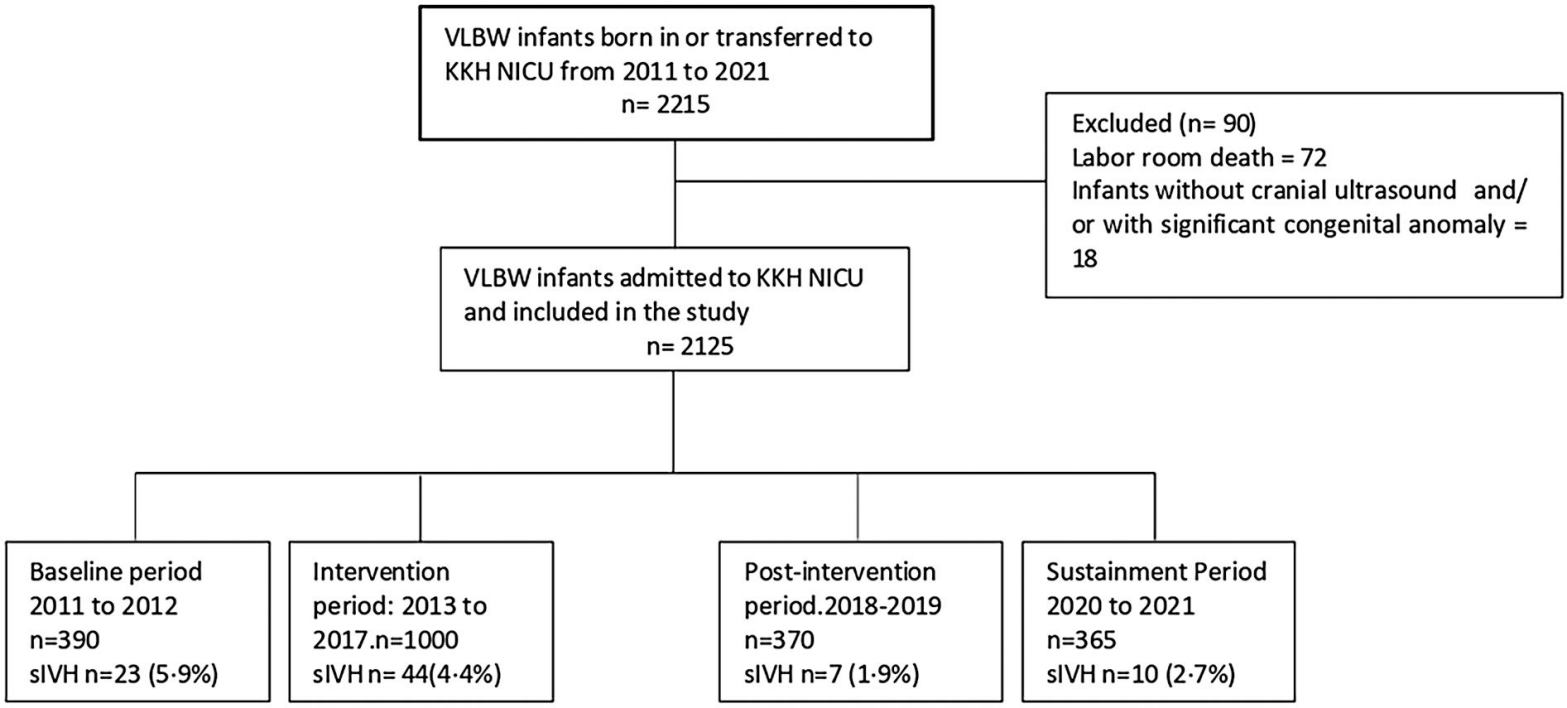
Consort flow diagram illustrating total number of infants in the study period, exclusion criteria, and excluded infants and final number of infants across four consecutive study periods.

All VLBW infants born between 2011 and 2021 at our center were categorized into four periods and included in the study (Figure 1). The mean SIVH rate from January 2011 to December 2012 (24 months) was considered representative of the baseline rate. The intervention period (2013 to 2017) was divided into two phases: Phase 1 (24 months), representing the pre-RC-PC-S period, and Phase 2 (36 months), representing the post-RC-PC-S period. The post-intervention period (2018–2019, 24 months) was deemed sufficient to evaluate the outcomes of the interventions, aligning with the original SMART aim of the study. Due to resource constraints, data for the sustainment period were limited to 18 months

Comprehensive data was collected from infants born during the entire study period(all four cohorts). Infants with significant congenital anomalies and labour room deaths were excluded from the study. The RC-PC-S recommendations were communicated to the department during the intervention period and afterward (Supplementary Material S1, Table S4).

### Methodology: salient definitions, measures, data collection, and statistics

The study period was characterized in to four phases as described previously. The modified QI methodology and IVH prevention bundle are deemed successful if the intervention leads to ≥50% risk reduction for severe IVH in the post intervention period. Maternal and infant characteristics were captured from a prospectively collected database. Some data about SIVH associations were captured retrospectively from electronic medical records of the mother and infant.

### Outcome measures

Patients were identified using the record kept in the NICU, updated every 24 h. This study included any live inborn or outborn neonate delivered or transferred to the NICU with a birth weight of ≤15 kg, which identified study population for data collection.

The primary outcome was SIVH, defined as any grade 3 or 4 IVH diagnosed on head ultrasound per the Papile definition^2^ Venous infarcts are also categorized as severe IVH. SIVH was diagnosed with a cranial ultrasound scan performed by the radiologist and POCUS (point of care ultrasonography) results by the managing team. The ultrasound examination was routinely performed by radiologists on Day 1,3, 14, and 28, and at term gestation in extremely low birth weight (≤1 kg, ELBW) infants and Days 1, 3, 28, and term in VLBW infants weighing between ≥I kg to 1·25 kg birth weights. Day 3 and term scan were performed for infants weighing between >1·25 and 1·50 kg. Experienced pediatric radiologists interpreted all radiologist-performed scans. There were no significant changes in the interpretation during the study period. In addition, the managing team performed POCUS scans, and SIVH captured in those scans before 28 days of life is also included. The data also includes SIVH cases, which could have happened before birth unless the bleed was demonstrated in the antenatal scans. The neonatal mortality rate was included as a secondary outcome measure.

We monitored several process measures based on adherence to key interventions. Compliance with all eight key interventions was measured, starting with adherence to the best practice recommendations for neutral head position and developmental care. Six key subsequent interventions followed as described under methodology. Process control charts were plotted for the rates of Hypothermia and antenatal magnesium administration.

Our balancing outcomes included global outcomes for VLBW infants, as described in Table 2, Infant characteristics. The outcomes were defined using the Vermont Oxford data manual of Definitions 2019. Key outcome definitions are also provided in Table 2.

### Analysis

Data were interpreted using a statistical process control chart created in Excel. Control limits were set at three standard deviations from the mean, and the central line was shifted when sustained special cause variation was established. Six monthly SIVH rates were plotted to demonstrate a clinically meaningful outcome, as the numbers were low. Three monthly rates were plotted to establish special cause variation for the death rate and process control charts. Characteristics between the groups were analysed with two-tailed *t*-tests or X^2^ tests where appropriate. *P*-values of <0.05 were considered statistically significant. Multivariate analyses were also performed.

## Results

**Figure 2:**
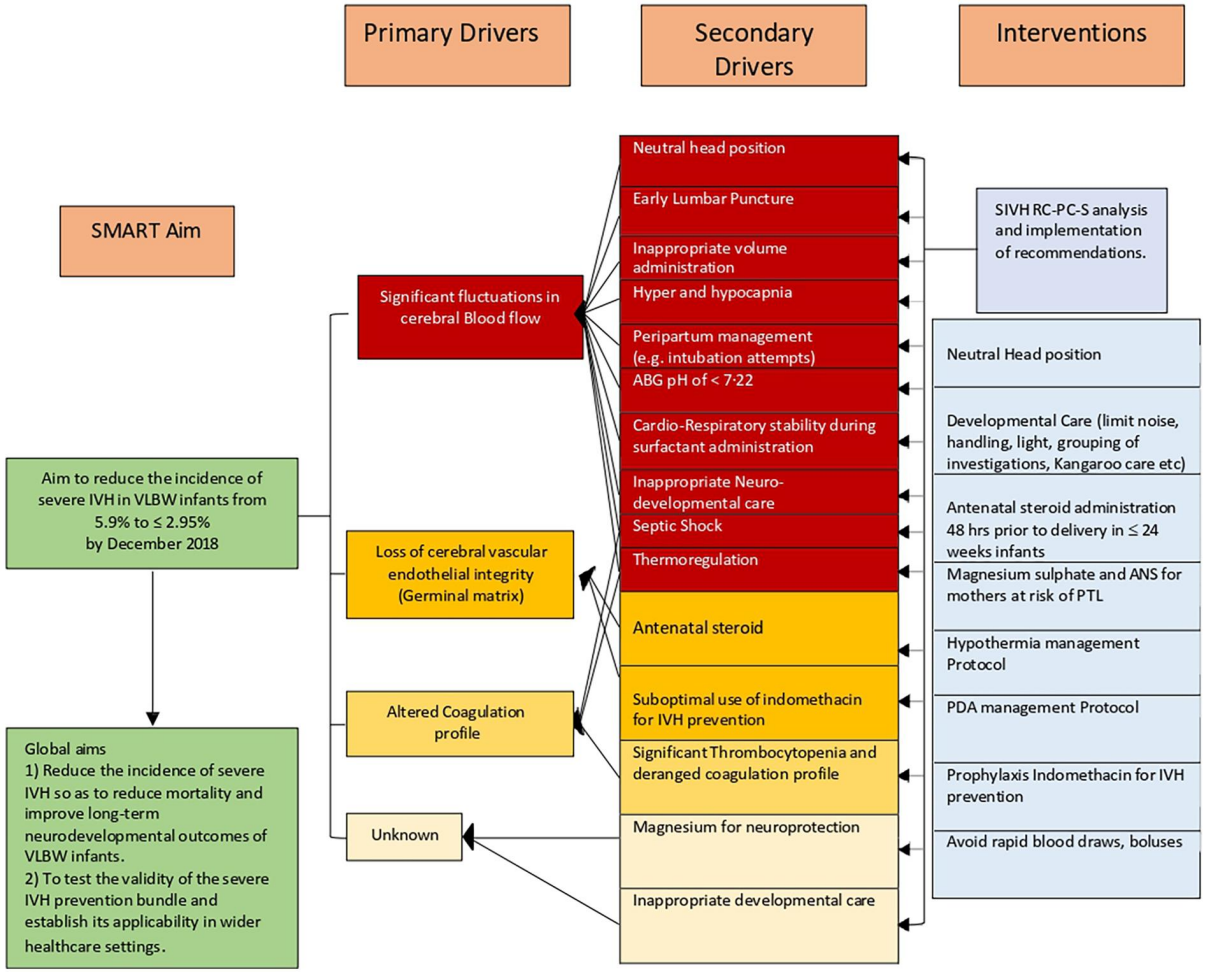
Key driver diagram for severe IVH prevention. ABG, arterial blood gas; ANS, antenatal steroid administration; PTL, preterm labour; PDA, patent ductus arteriosus; SIVH, severe intraventricular haemorrhage; SMART, specific, measurable, applicable, realistic, and timely, RC-PCS Root Cause Process Compliance System analysis.

During the study period from 2011 to 2021, 2,215 VLBW infants were born. The study excluded ninety infants (Figure 1). There was no difference in most of the baseline maternal and infant characteristics (Table 1 & 2). The birth weight (mean 1.08 kg, SD 0.26 kg) and gestational age (mean 29.5 weeks, SD 2.8 weeks) during the pre-intervention period were similar to those observed during the intervention, post-intervention, and sustainment periods. In the sustainment period, the mean birth weight was 1.08 kg (SD 0.30 kg), and the mean gestational age was 28.9 weeks (SD 2.87 weeks). The number of infants with an Apgar score < 5 at 5 min and the CRIB II score was comparable across the periods. In the post-intervention period, fewer mothers received antenatal steroids and tocolytic therapy. There was a significant improvement in the primary outcome of the rate of SIVH. The incidence of SIVH was 5·9% in the pre-intervention period. Special cause variation was achieved within 2018, resulting in a reduction of SIVH from 4·4% to 1·9% post-intervention (*p* = 0·006) (Figure 2), representing a reduction of 57%. The incidence of SIVH was 2·7% in the sustainment period. In the multivariate analysis, with the model including gestational age (weeks), Plurality, Place of delivery, Tocolysis, and Sepsis, the risk of SIVH was reduced in the post-intervention period by 74% [adjusted OR 0·266(0·103,0·687),*p* = 0·0063]. The secondary outcome of neonatal mortality demonstrated a decreasing trend, declining from 5.4% to 3% in the post-intervention period (*p* = 0.067).

**Table 1:** Summary of maternal characteristics.

Variable	Baseline period	Intervention Period	Post Intervention Period	*P*-value (Intervention period compared to post intervention period	Sustainment Period	*P*-value (Comparison across the four periods)
	2011–2012 (*n* = 390)	2013–2017 (*n* = 1000)	2018–2019 (*n* = 370)		2020–2021	
Maternal age (years), mean (SD)	31.3 (5.65)	32.2 (5.28)	33.1 (4.94)	0.0022	33.2 (5.03)	0.001
Multiple pregnancies, *n* (%)	118 (30.3)	258 (25.8)	71 (19.3)	0.0125	73 (20.1)	0.0006
Caesarean section, *n* (%)	262 (67.2)	702 (70.2)	266 (72.3)	0.5078	253 (69.3)	0.0865
Fertility treatment, *n* (%) (Assisted Conception)	57 (14.6)	180 (18.0)	47 (12.8)	0.0212	55 (15.1)	0.0855
Maternal smoking, *n* (%)	23 (5.9)	26 (2.6)	5 (1.4)	0.1713	6 (1.6)	0.0004
Amniocentesis, *n* (%)	50 (12.9)	84 (8.4)	16 (4.3)	0.0107	17 (4.7)	0.0001
Tocolytic therapy, *n* (%) (tocolysis)	190 (48.7)	476 (47.7)	87 (23.7)	< 0.0001	57 (15.6)	0.0001
Pregnancy-induced hypertension (PIH), *n* (%)	104 (26.7)	255 (25.5)	108 (29.3)	0.1558	98 (26.8)	0.5674
Premature rupture of membranes (PROM), *n* (%)	113 (29.0)	267 (26.7)	83 (22.6)	0.1192	66 (18.1)	0.0015
Chorioamnionitis (clinical & biochemical), *n* (%)	146 (37.4)	332 (33.2)	101 (27.4)	0.0424	113 (31.0)	0.0266
Antenatal steroids, *n* (%) None/incomplete (A/N Steroid)	111 (28.5)	301 (30.1)	164 (44.6)	<.0001	83 (22.7)	0.0001
Child birth before arrival in Hospital, n(%) (Place of delivery)	2 (0.5)	17 (1.7)	8 (2.2)	0.5617	5 (1.4)	0.2619

**Table 2:** Summary of infant characteristics.

Variable	Baseline period	Intervention Period	Post Intervention Period	*P*-value (Intervention period compared to post intervention period	Sustainment Period	*P*-value (Comparison across the four periods)
	2011–2012	2013–2017	2018–2019		2020–2021	
	(*n* = 390)	(*n* = 1,000)	(*n* = 370)		(*n* = 365)	
Birth weight (kg), mean (SD)	1.08 (0.26)	1.07 (285.0)	1.11 (271.2)	0·035	1.08 (0.30	0.1971
Gestational age (wk), mean (SD)	29.1 (2.8)	28.6 (2.92)	28.8 (2.73)	0.390	28.9 (2.87)	0.037
Gestational age, median (range)	29.0 (23, 39)	29.0 (23, 38)	29.0 (22, 36)	0.303	28.9 (23, 37)	0.663
Small for gestational age *n*,(%)	139 (35.6)	300 (30·0)	114 (31·0)	0·725	97 (26.6)	0.001
Gender, *n* (%) Male	198 (50.8)	508 (50·8)	176 (47·6)	0·288	180 (49.3)	0.7310
Ethnicity, *n* (%)						0.014
Chinese	209 (53.6)	543 (54·3)	186 (50·3)	0·184	204 (55.9)	
Indian	45 (11.5)	98 (9·8)	38 (10·3)	0·553	27 (07.4)	
Malay	96 (24.6)	253 (25·3)	83 (22·4)	0·776	90 (24.7)	
Others	40 (10.3)	106 (10·6)	63 (17·0)	0·002	16 (12.1)	
Apgar score < 5 at 5 min, *n* (%)	34 (8.87)	69 (6.87)	21 (5.70)	0.438	16 (4.51)	0.099
CRIB II score mean (SD)	6.80 (3.518)	5.46 (3.74)	6.25 (3.48)	0.000	6.50 (3.81)	0.331
Airleaks, *n* (%)						0.0103
Pulmonary interstitial emphysema	4 (1.0)	16 (1·6)	2 (0·5)	0·564	1 (0.3)	
Pneumothorax	18 (4.7)	29 (2·9)	6 (1·6)	0·183	4 (1.1)	
Pulmonary haemorrhage, *n* (%)	16 (4.1)	28 (2·8)	4 (1·1)	0·073	2 (1.3)	0.0485
Chronic lung disease (CLD) (O_2_ dependency at 36 weeks), *n* (%)	65 (18.1)	255 (27·1)	104 (29·5)	0·392	122 (37.2)	0.001
Necrotising enterocolitis (NEC), *n* (%)						
NEC ≥ stage 2	21 (5.6)	18 (1·8)	3 (0·8)	0·738	9 (2.5)	0.0044
Solitary GI perforation/spontaneous intestinal perforation (SIP)	6 (1.6)	23 (2·3)	5 (1·3)	0·271	4 (1.1)	
Sepsis, *n* (%)						0.0858
Acquired	40 (10.6)	68 (6·8)	18 (4·8)	0·189	20 (5.6)	
Early onset (<72 h)	8 (2.1)	19 (1·9)	8 (2·1)	0·351	7 (2.0)	
Retinopathy of prematurity (ROP), *n* (%)						
≥Stage 3	24 (7.2)	60 (6·0)	14 (3·8)	0·116	49 (14.2)	0.011
Duration of continuous positive airway pressure (CPAP) (days)						
Mean (SD)	23.2 (33.6)	22.6 (31.8)	22.9 (28.9)	0.8604	29.3 (31.9)	0.1329
Median, Range	8.0 (0, 252)	9.0 (0, 221)	10.0 (0, 184)	0.1384	18.0 (0, 188)	0.0012
Duration of intubation (days)						
Mean (SD)	31.2 (44.4)	31.7 (45.1)	28.5 (36.5)	0.1859	38.6 (51.9)	0.0636
Median, Range	10.0 (0, 352)	12.0 (0, 301)	12.0 (0, 191)	0.5697	20.0 (0, 365)	0.0017
Duration of neonatal intensive care unit (NICU) stay (days),						
Mean (SD)	43.7 (48.6)	42.8 (46.4)	37.7 (39.0)	0.0421	49.2 (56.5)	0.0130
Median, Range	29.5 (0, 352)	26.0 (0, 365)	22.5 (1, 191)	0.0087	29.0 (0, 417)	0.0399

Overall, the VLBW mortality rate inclusive of all VLBW infants admitted to the NICU was 11.5%, 12·5%, 8·6%, and 7.3 percent in the preintervention, intervention, post-intervention, and sustainment periods, respectively.

**Figure 3:**
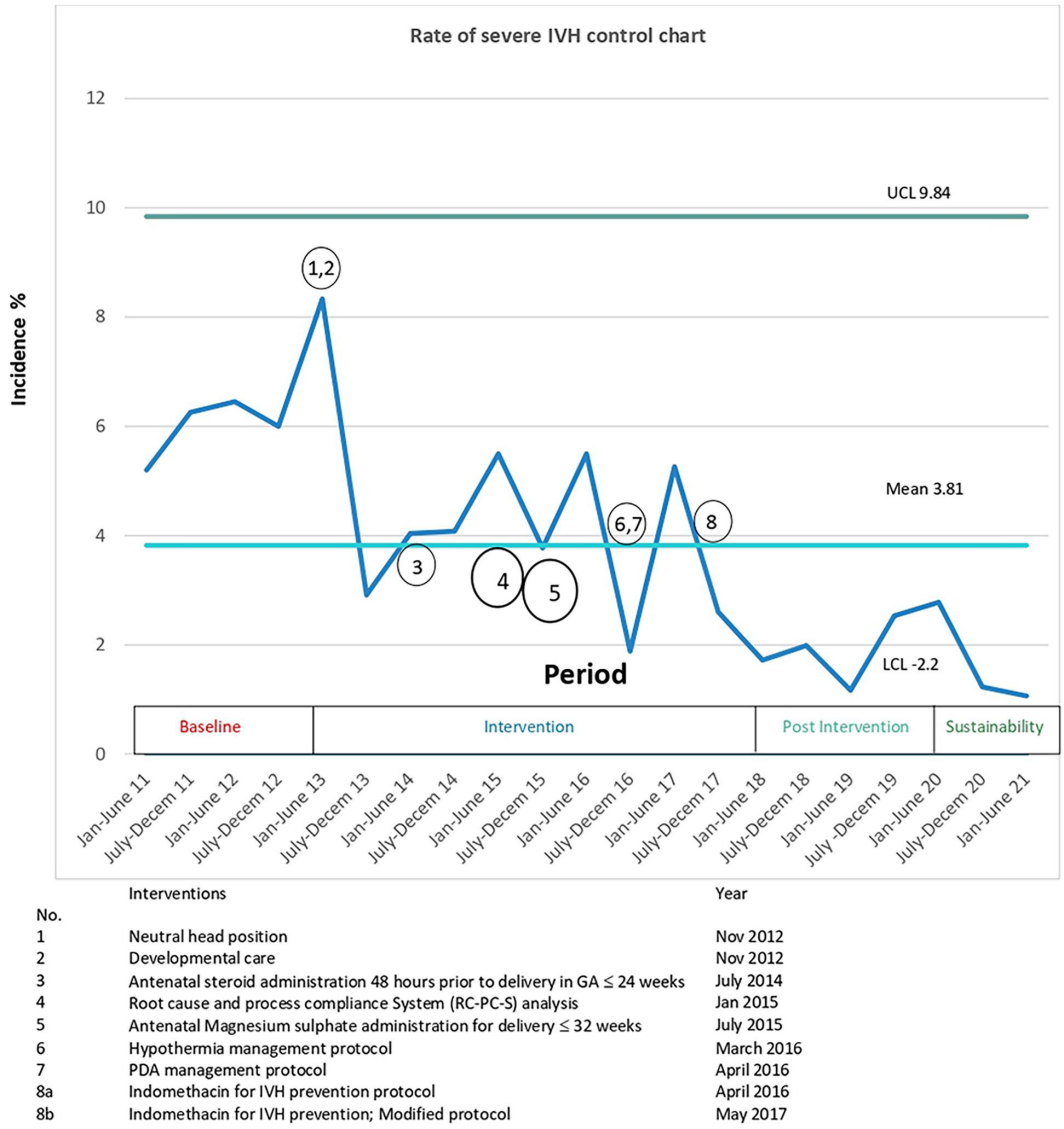
Control chart for the rate of SIVH, grouped by 6 monthly incidences and annotated with interventions. The mean rate of sIVH decreased from 5.9% to 1.9% and special cause rule of 8 points below the center line was met in first half of 2021 (50). CL, Upper control limit; LCL lower control limit; SIVH, severe Intraventricular hemorrhage.

The compliance rate to process measures was above 80% for all seven eight interventions apart from the routine use of antenatal magnesium sulfate administration (56%) in the intervention period (Supplementary Material S1, Table S7), which can be explained by delayed introduction on the obstetric side. There were differences in the balancing measures. Chronic lung disease significantly increased across the study periods (starting from the baseline period). Stage 2 necrotizing enterocolitis showed a reduction followed by an increase during the sustainment period, while Stage 3 retinopathy of prematurity increased in the same period (Table 2). Pulmonary hemorrhage decreased from 4.8% to 1.3% in the sustainment period (*p* = 0.048). The proportion of SGA infants gradually declined across the study periods (from the baseline period onward). The duration of CPAP, intubation, and intensive care unit stay followed a similar trend. The control charts met special cause rules for SIVH, mortality(Outcome), and hypothermia incidence and antenatal magnesium sulfate administration(Process) (Figure 3 and Supplementary Material S2, Figure S4–S6).

## Discussion

The team implemented a bundle of evidence-based neuroprotective strategies and novel RC-PC-S process for the care of very low-birth infants, including developmental care, hypothermia prevention protocol, and consistent and optimal use of antenatal dexamethasone and indomethacin prophylaxis; the project significantly reduced the rate of severe IVH by 57%, from 4·4% to 1·9%, with sustained improvement over the next two years. The mortality was reduced during this period. This reduction was accomplished without increasing markers of other morbidity in this at-risk patient population.

### Interpretation

#### Summary

SIVH is a strong risk factor for both death and adverse neurodevelopmental outcomes in preterm infants. Although our baseline data compared favourably with those in other centres in the VON network, being in the 5·9%, best quartile, this project focussed on s IVH was embarked upon in recognition of the potential to reduce this morbidity as well as death and neurodevelopmental delay. Mixed success has been achieved with initiatives aimed to reduce SIVH in preterm infants (33) elsewhere but QI work focused on delivery room management and bedside neuroprotective strategies has been shown to produce significant improvement in some centres (14, 34, 35).

#### Analysis

The team acknowledged the project's complexity after an increase in SIVH rate was seen in 2015 following a decline in 2014. The initial interventions were reinforced with RC-PC-S analysis of individual cases, and recommendations in line with best practices were implemented. The key objectives of the Root cause and process compliance analysis were to identify the direct and indirect factors contributing to the development of severe intraventricular hemorrhage (IVH), including system-related issues and upstream causes. This approach, in addition to recognizing the known pathophysiological causes of IVH, identifies factors not directly linked to its pathogenesis, for example, delayed delivery in the context of preterm rupture of membranes(PROM) and gram-negative urinary tract infection in the mother, can lead to clinical chorioamnionitis and gram-negative septic shock and severe IVH in extremely low birth weight infants. RC-PC-S analysis also evaluates the compliance to processes established to prevent severe IVH, ensuring process compliance, adherence to standard practices and identifying areas of improvement. The application of the RC-PC-S process is not limited to any period as it is integrated into the routine evaluation of infants who develop any significant morbidity without the need for additional workforce or resources. The RC-PC-S process serves as a valuable tool for identifying new interventions and can be sustained as a QI tool over an indefinite period. This study formulated ten recommendations using this approach, highlighting the efficacy of the process. Five (i, iii, v, vi, ix, x) of ten recommendations were linked to best practice (19), of which hypothermia prevention(vi) was implemented as a structured protocol (25). Hypocarbia was a frequent finding in extremely low birth weight infants (ELBW) who were ventilated with high-frequency oscillatory ventilation (HFOV). In an observational study of 1,125 ELBW infants, HFOV was associated with a higher incidence of SIVH, and the audit team recommended restricting it's use in ELBW infants. Some centres reported good results with high-frequency jet ventilation in small infants. This mode of ventilation is not available in our unit. Septic shock requiring significant inotropic support is also a known risk factor for SIVH so reducing the incidence of early and late-onset sepsis can potentially reduce the incidence of SIVH. Deep endotracheal tube and delayed diagnosis of airway malformation (a rare anomaly) can potentially lead to pneumothorax and hypercarbia, respectively, thereby potentially influencing the s IVH incidence. Overall, this modified QI approach may be associated with reducing SIVH to 1·9% in VLBW infants.

A sustained improvement in the primary outcome was not attained until three years into the project. This was attributed to a late response to the cumulative effect of interventions reaching a threshold effect on SIVH causation, the impact of sustained education, the introduction of the SIVH audit, and subsequent implementations of recommendations. The other key factor that led to the late improvement was the role played by the nurses and allied health professionals including the RT's in educating and monitoring the implementation of the interventions in the intensive care unit. The team recognises the importance of the RC-PCS process, and we recommend serious attention be given to this by other units looking to implement similar projects. We acknowledge that the outcome of this project could have been influenced by other policy changes in the unit only in the post-intervention and sustainment study period. The PremFirst Hour (or Neonatal Golden Hour) (36, 37) project was implemented after the intervention phase and is unlikely to have influenced the outcomes, as its key component, relevant to severe IVH—temperature regulation—had already been addressed through the SIVH prevention bundle. Following the post-intervention period, the unit initiated a non-invasive surfactant administration project, and a revised CPAP policy was introduced. Both interventions are supported by established evidence and are expected to lower the risk of severe IVH, alongside other benefits. Notably, although less frequently highlighted, there is clear evidence that failure of CPAP or non-invasive surfactant therapy is associated with a substantially increased risk of severe IVH and other morbidities, particularly in infants born at less than 28 weeks' gestation—the specific population targeted for severe IVH prevention. The unit offered CPAP to infants of all gestational ages, and non-invasive surfactant was offered to infants ≥ 25 weeks of gestation. Based on a limited RC-PC-S analysis, the authors concluded that these changes, implemented after the post-intervention phase, may have negatively impacted the unit's severe IVH burden (38–41). Additionally, a delayed cord clamping (DCC) policy was introduced in 2023. An analysis of 996 infants with a gestational age ≤28 weeks, drawn from three studies, showed that while DCC was associated with reduced mortality, it had no significant effect on the incidence of severe IVH (42).

The project design did not include a cost-benefit analysis plan. However, our interventions did not increase the cost of patient care. The selective PDA treatment protocol (29) reduced the use of Cox inhibitors in VLBW infants. The local listing prices of a vial (2 mmol/ml 5 ml) of magnesium sulphate and an ampoule of dexamethasone (4 mg/ml) are less than five USD respectively.

Our findings are consistent with those of similar studies (18, 43, 44). A recent review identified 14 studies reporting on severe intraventricular hemorrhage (IVH), most of which originated from North America or Europe. Of these, nine studies showed improvements in severe IVH outcomes. Eight studies reported adjusted odds ratios (OR) ranging from 0·32 to 1·23. Five studies published process control charts, though only one made reference to SQUARE guidelines. All studies were part of time-limited quality improvement (QI) initiatives, with no provisions to integrate all QI components into routine management practices. Furthermore, many studies may have failed to capture all cases of severe IVH due to early deaths or standardized cranial scan protocols. The current study offers evidence from the largest cohort of infants (*n* = 1,735), with this report meeting all key quality indicators, including process run charts, adjusted odds ratios (AOR), and adherence to SQUIRE guidelines. This study reported an AOR of 0·266 for severe IVH in the post-intervention period, the lowest in the literature. The accuracy of severe IVH reporting was enhanced by including cases identified via point-of-care ultrasound (POCUS). Additionally, the study introduced a novel RC-PC-S analysis as part of the QI process.

Careful analysis of outcomes is essential when introducing interventions for VLBW infants.

Morbidities in VLBW infants are interconnected with the possibility that improvement in one morbidity potentially adversely affects other outcomes (45, 46). Since the implementation of this project, there have been no significant negative changes in balancing measures up to the sustainment period. The new policies discussed earlier explain the negative changes in the balancing measures observed during the sustainment period. The changes in balancing measures related to respiratory morbidities and NICU stay are not related to the study interventions.

The sustainability of the outcome requires continued effort. In addition to monitoring the implementation of current interventions, the RC-PC-S process may identify new issues that require intervention, such as the prevention of early-onset sepsis in infants of mothers with PPROM, which aims to prevent SIVH and will necessitate close collaboration with obstetricians.

### Limitations

This study has limitations. Continuous monitoring of adherence was carried out for hypothermia prevention and antenatal magnesium chloride administration during the intervention and post-intervention periods. In contrast, other interventions were tracked at 6- to 12-month intervals. Among these, compliance with neutral head positioning and developmental care was monitored for the shortest duration, with tracking discontinued after six months once satisfactory adherence was observed. The authors acknowledge that this approach may have compromised the sustained effectiveness of these interventions over time. Addressing this limitation was one of the key objectives of the RC-PC-S analysis, both during the project and in the post-implementation phase.

The cumulative nature of the effect of interventions on the outcome makes it difficult to determine the degree to which each factor contributed to the observed improvement in outcome, and the study did bring out this issue as a problem to address. The authors plan to design a future study aimed at evaluating the specific impact of each individual intervention within the bundle on the prevention of severe IVH (47). As is the inherent nature of all QI initiatives, improvements in outcomes are associations, not causations.

The use of indomethacin for preventing severe intraventricular haemorrhage (IVH) is complex, involving considerations such as the rate of severe of IVH, potential adverse effects of the medication, and long-term neurodevelopmental outcome. The authors recommend its use only in carefully selected, very high-risk infants (47, 48).

Finally, the Potential associated improvement in the neurodevelopmental outcome of the cohort can only be determined by long-term developmental follow-up, as IVH is only one of the risk factors for poor neuro-developmental outcomes (49).

## Conclusions

Implementing the SIVH prevention bundle, characterized by optimal use of indomethacin prophylaxis, antenatal betamethasone, and magnesium sulfate for fetal neuroprotection and the RC-PC-S process, is associated with reduced SIVH in VLBW infants. Monitoring of long-term neurodevelopmental outcomes is recommended.

## Data Availability

The raw data supporting the conclusions of this article will be made available by the authors, without undue reservation.
