# Supplementary material for: A sustained reduction in the rate of severe intraventricular hemorrhage in very low birth weight infants: a novel quality improvement project in a large perinatal-neonatal centre in Asia
Source: Front Pediatr. 2025 Sep 29;13:1640964. doi: 10.3389/fped.2025.1640964 (PMC12515680; doi:10.3389/fped.2025.1640964)
Supplement: Supplementary file 1 [file Supplementaryfile1.docx]

**Supplementary material 1**

**Record of RC-PC-S Analysis and Recommendations and**

**Description of Selected Severe IVH Prevention Bundle Interventions.**

Table3a.Root Cause-Process Compliance-System Analysis (RC-PC-S Analysis) outcome document for severe IVH

| **S/N** | Root Cause (RC-) | Y/N | Process Compliance (PC) | Y/N | System Analysis (-S) | #A/B/C | Recommendations |
| --- | --- | --- | --- | --- | --- | --- | --- |
| 1 | Lack of complete antenatal steroids ^1^  (See definitions below) |  | Compliance with  O&G protocol |  | Leaning environment   1. Foster learning (Support, respect engagement 2. Neutral 3. Hinders Learning   Leadership   1. Selection 2. Development 3. Delivery   Attitude of the team  A) Driving Change Dynamics  B) Neutral  c) Resistance  Training & accreditations  Basic training: A) Theoretical knowledge  B) Practical skills  Advanced training  A)  Theoretical knowledge  B) Practical skills  Interdepartmental policy or coordination domain issues  Interhospital or  national level  policy or coordination domain issues  Legislature domain issues |  | (Please see Supplementary material 1. Table 5) |
| 2 | Lack of Optimal resuscitation ^2^ |  | Compliance with  Singapore NRP Guideline |  |  |  |  |
| 3 | Infant requiring significant resuscitation^3^ |  | Compliance with  Singapore NRP Guideline |  |  |  |  |
| 4 | Lack of Indomethacin prophylaxis in first 24 hours of life, when appropriate |  | Compliance with Neonatal Dept Guideline |  |  |  |  |
| 5 | Lack of Antenatal MgSO4 |  | Compliance with  O&G protocol |  |  |  |  |
| 6 | Mode of Delivery: Vaginal |  | Compliance with established literature/O&G protocol on mode of delivery |  |  |  |  |
| 7 | Lumbar Puncture within first 72 hours of life |  | Compliance with established QI recommendation and literature |  |  |  |  |
| 8 | Fluid boluses given without overt hypotension ^5^ |  | Compliance with established QI recommendation and literature |  |  |  |  |
| 9 | Chest physiotherapy within first 72 hours of life and routine suctioning |  | Compliance with established QI recommendation |  |  |  |  |
| 10 | Hypocarbia or hypercarbia within first 72 hours of life ^6^ |  | Compliance with established QI recommendation and literature |  |  |  |  |
| 11 | Pneumothorax (or any air leaks) |  | Compliance with established QI recommendations and literature |  |  |  |  |
| 12 | Significant hypotension needing inotropes |  | Timely recognition of hypotension, etiology and timely management |  |  |  |  |
| 13 | Pulmonary haemorrhage |  | Compliance with PDA treatment protocol and PH treatment dept policy |  |  |  |  |
| 14 | Accidental extubation or difficult resuscitation with first 72 hours of life |  | Compliance with ET securing and monitoring policy and S NRP guideline |  |  |  |  |
| 15 | Difficult procedure within the first 72 hours of life ^7^ |  | Compliance with Severe IVH prevention QI recommendation |  |  |  |  |
| 16 | Significant PDA requiring treatment |  | Compliance with department guideline |  |  |  |  |
| 17 | Rapid infusion of Sodium Bicarbonate ^8^ |  | Compliance with Severe IVH prevention QI recommendation |  |  |  |  |
| 18 | Significant coagulopathy / thrombocytopenia ^9^ |  | Compliance with Severe IVH prevention QI recommendation on correction of abnormality |  |  |  |  |
| 19 | Lack of delayed cord clamping |  | Compliance with protocol |  |  |  |  |
| 20 | Summary  Preventable or not? |  |  |  |  |  |  |

# A = Appropriate

# B = Need evaluation and follow up action.

# C = Not assessed

Table3b.Record of Root Cause-Process Compliance-System Analysis (RC-PC-S Analysis) and outcome document for Hypothermia prevention in very low birth weight infants.

| **S/N** | Root Cause (RC-) | Y/N | Process Compliance (PC-) | Y/N | System Analysis  (S) | #A/B/C | Recommendations |
| --- | --- | --- | --- | --- | --- | --- | --- |
| 1 | Delivery and OT Environ temp not ≥ 24 ^o^C | N | Compliance with current hypothermia prevention protocol | Y | Leaning environment   1. Foster learning (Support, respect engagement 2. Neutral 3. Hinders Learning | C | 1.Update the existing hypothermia prevention protocol to comprehensively address root causes, compliance gaps, and systemic challenges. This includes enhancing interdepartmental coordination—particularly with Obstetrics and Gynaecology (O&G), ensuring availability of a portable battery-powered transport incubator for NICU transfers, installing additional heat sources in the operating theatre, and resolving procurement delays related to chemical heating mattresses. Leadership engagement and regulatory alignment are also critical to mitigate the risk of hypothermia in very low birth weight (VLBW) infants.  2. **Implement a Hypothermia Management Protocol** Develop and introduce a standardized protocol for the early recognition and clinical management of hypothermia in neonates, with a focus on timely intervention, documentation, and outcome monitoring. |
| 2 | Appropriate application of Polyethylene bag ≤ 32 weeks infants | N | Compliance with current hypothermia prevention protocol | Y |  |  |  |
| 3 | Application of preheated chemical mattress for resus and Procedures | N | Compliance with current hypothermia prevention protocol | Y | Leadership   1. Selection 2. Development 3. Delivery | B |  |
| 4 | Appropriate application of Woolen Cap and prewarming of Linen | y | Compliance with current hypothermia prevention protocol | Y | Attitude of the team  A) Driving Change Dynamics  B) Neutral  c) Resistance | A |  |
| 5 | Measurement of infants Temp at key points during resus , transport and admission to NICU | N | Compliance with current hypothermia prevention protocol | Y | Training & accreditations  Basic training: A) Theoretical knowledge  B) Practical skills  Advanced training  A)  Theoretical knowledge  B) Practical skills | C |  |
| 6 | Over head heater and power source during transport from DS to NICU | N | Compliance with current hypothermia prevention protocol | Y | Interdepartmental policy or coordination domain issues | B |  |
| 7 | Use of overhead heater on 100% power when repeated or prolonged opening of incubator doors required for care/procedure | N | Compliance with current hypothermia prevention protocol | Y | Interhospital or  national level  policy or coordination domain issues | B |  |

# A = Appropriate

# B = Need evaluation and follow-up action

# C = Not assessed

**Table 4. List of severe IVH prevention RC-PC-S analysis recommendations.**

| No | **Recommendations** |
| --- | --- |
| i | Optimize the ventilator mode and settings so as to avoid hyper and hypercapnia in the initial 7 days of life in VLBW infants |
| ii | Optimize the ET tube position at the earliest so as to avoid pneumothorax and inadvertent surfactant administration to right lung |
| iii | Liaise with the obstetrician to consider administering second course of antenatal steroid if first course was administered >14 days before the delivery. |
| iv | Implement the recommendations of the sepsis QI team |
| v | All infants born with gestational age <26 week and with incomplete or no antenatal steroid doses, should receive prophylactic dose of indomethacin to prevent IVH (0·1mg/kg/day 24 hourly) for 3 doses. |
| vi | Establish temperature regulation for VLBW infants at birth to target a temperature of 36·5 degree Celsius on admission to the NICU. |
| vii | Early diagnosis of airway malformation in VLBW infants by referring and performing micro laryngobronchoscopy. |
| viii | Restrict the use of HFOV in ELBW infants and its use is recommended only as a life saving measure^29^. |
| ix | To have comprehensive evaluation of contraindication of COX inhibitors for treatment of PDA in high risk infants and consider its use after evaluation and weighting the pros and cons of treatments. |
| x | Keeping a high index of suspicion for necrotizing enterocolitis in infants with feed intolerance, worsening metabolic acidosis and suspicious abdominal X-rays and optimal management should be initiated |

**Table 5.List of practices to enhance neurodevelopmental care**

| **Minimise environmental stimulation** |
| --- |
| Established pre-set twice daily “quite time” where infants were offered complete rest by avoiding environmental and care related stimulation( handling, no routine suctioning in 72 hrs, painful procedure, active control of light and noise) |
| Established noice sensors throughout the nursery |
| Established pre-set “touch times”(Clubbing of investigations, procedures and handling |
| **Minimise Blood pressure lability** |
| A.Avoid rapid blood draws and flushes B. Avoid peripheral blood measurement when artery catheter present |

**Table 6.Key features of hypothermia prevention protocol**

| 1.Open mode transport on Giraffe Omnibed incubator with shuttle battery |
| --- |
| 2.Giraffe Omnibed incubator was kept in open mode while performing the procedures |
| 3.Polyethylene wrap were removed only after the end of all the procedures during admission |
| 4.Modified the polyethylene wrap design and application in the NICU |
| 5.Application of appropriate size woollen bonnet during resuscitation |
| 6.Documenting normal temperature before leaving the delivery suite |
| 7. Delivery room and NICU environmental temperature was kept at 24^o^C or above |
| 8. Installed additional heaters in the delivery suite |
| 9.Staff(Physician’s, Nurses ,allied health professionals) were trained using simulated Video. |
| 10.Planned heating mattress use for procedures was delayed due to regulatory requirements. |

**Table 7. Compliance with Interventions**

| No | **Interventions** | Compliance rate (%) |
| --- | --- | --- |
| 1 | Neutral head position | 77·7 |
| 2 | Developmental care | 82·2 |
|  | Indomethacin for IVH prevention Protocol | 80 |
| 4 | Hypothermia protocol | 92·4 |
| 5 | Appropriate timing for antenatal steroid administration in mothers at risk of preterm labour and Foetus ≤ 24 weeks gestation as per QI recommendation | 97 |
