# Supplementary material for: A sustained reduction in the rate of severe intraventricular hemorrhage in very low birth weight infants: a novel quality improvement project in a large perinatal-neonatal centre in Asia
Source: Front Pediatr. 2025 Sep 29;13:1640964. doi: 10.3389/fped.2025.1640964 (PMC12515680; doi:10.3389/fped.2025.1640964)
Supplement: Supplementary file 2 [file Supplementaryfile2.pdf]

Supplementary material 2

Record of process compliance control charts

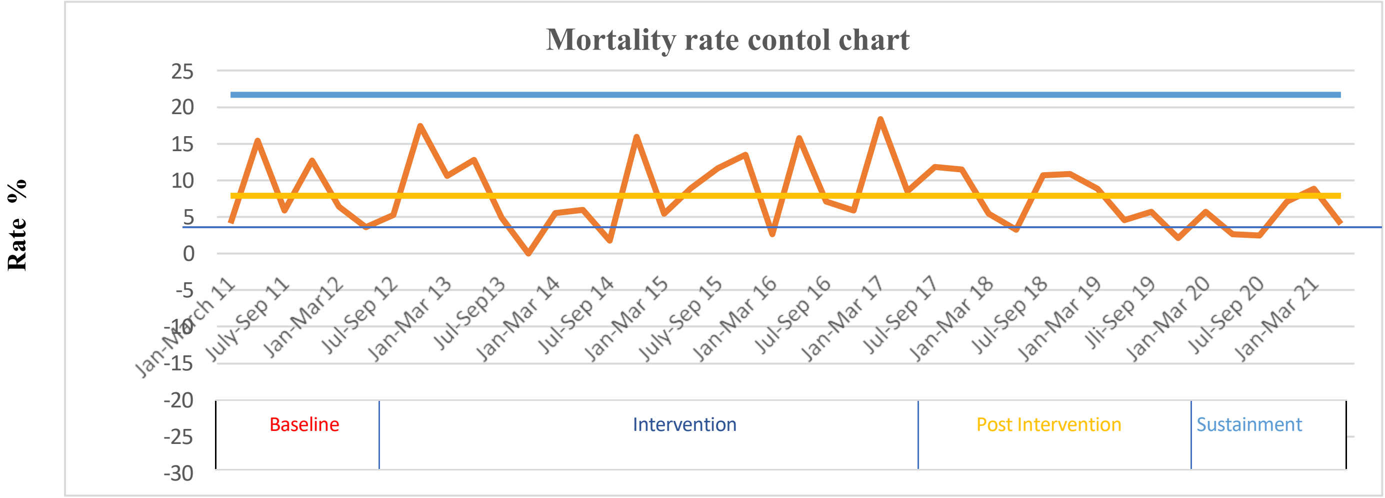

No.	Interventions	Year
1	Neutral head position	Nov 2012
2	Developmental care	Nov 2012
3	Antenatal steroid administration 48 hours prior to delivery in GA ≤ 24 weeks	July 2014
4	Root cause and process compliance System (RC-PCS) analysis	July 2015
5	Antenatal Magnesium sulphate administration for delivery ≤ 32 weeks	July 2015
6	Hypothermia management protocol	March 2016
7	PDA management protocol	April 2016
8a	Indomethacin for IVH prevention protocol	April 2016
8b	Indomethacin for IVH prevention; Modified protocol	May 2017

Figure 4
Control chart for the rate of mortality, grouped by 3 monthly rate and annotated with interventions. The rate of mortality decreased from 11·5 % to 7·3 % and special cause rule of 15 points within the mean ±I SD region was met in December of 2020(50).U CL , Upper control limit; LCL lower control limit.

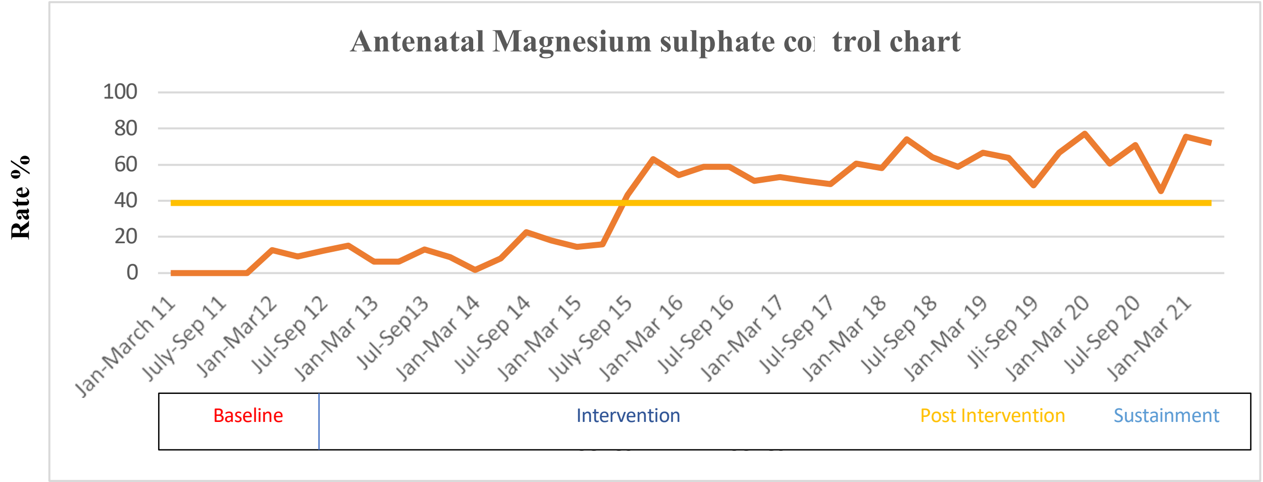

No.	Interventions	Year
1	Neutral head position	Nov 2012
2	Developmental care	Nov 2012
3	Antenatal steroid administration 48 hours prior to delivery in GA ≤ 24 weeks	July 2014
4	Root cause and process compliance System (RC-PCS) analysis	July 2015
5	Antenatal Magnesium sulphate administration for delivery ≤ 32 weeks	July 2015
6	Hypothermia management protocol	March 2016
7	PDA management protocol	April 2016
8a	Indomethacin for IVH prevention protocol	April 2016
8b	Indomethacin for IVH prevention; Modified protocol	May 2017

Figure 5

Rate of antenatal Magnesium sulphate administration control chart, grouped by 3 monthly incidences and annotated with interventions. The rate of magnesium sulphate administration increased from 12·7 % to 64·1% and special cause rule of 8 points above the center line was met in June 2017(50).U CL , Upper control limit; LCL lower control limit.

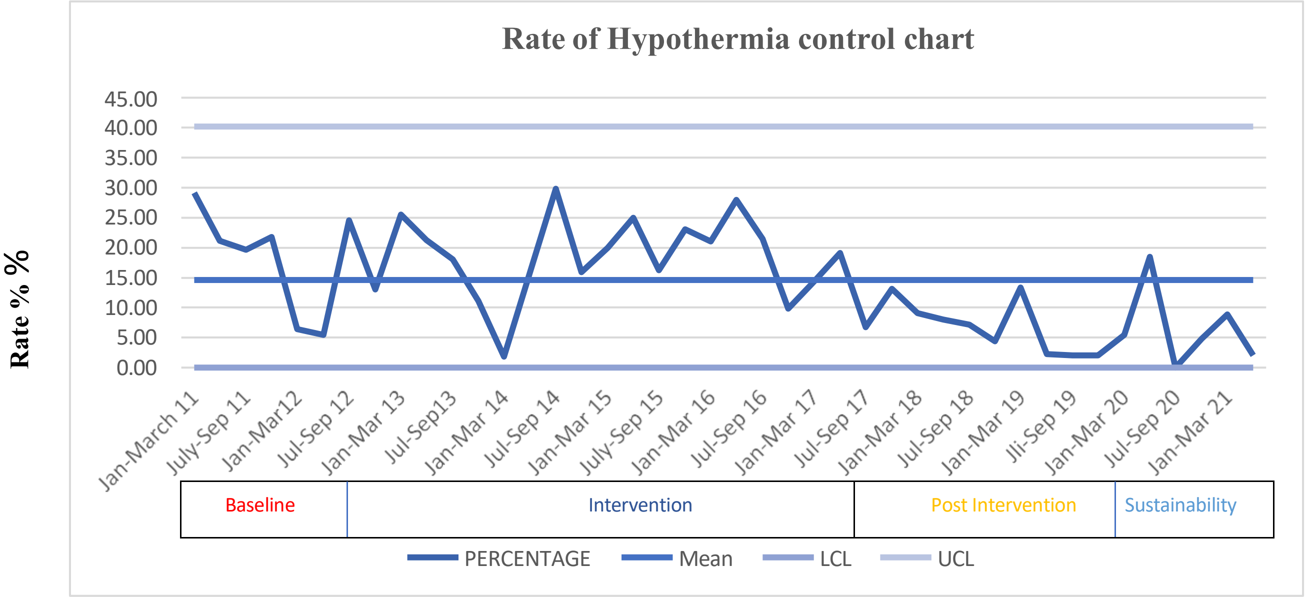

No.	Interventions	Year
1	Neutral head position	Nov 2012
2	Developmental care	Nov 2012
3	Antenatal steroid administration 48 hours prior to delivery in GA ≤ 24 weeks	July 2014
4	Root cause and process compliance System (RC-PCS) analysis	July 2015
5	Antenatal Magnesium sulphate administration for delivery ≤ 32 weeks	July 2015
6	Hypothermia management protocol	March 2016
7	PDA management protocol	April 2016
8a	Indomethacin for IVH prevention protocol	April 2016
8b	Indomethacin for IVH prevention; Modified protocol	May 2017

Figure 6
Control chart for the rate of Hypothermia, grouped by 3 monthly rate and annotated with interventions. The mean rate of hypothermia decreased from 21·05 % to 2 % and special cause rule of 8 points below the center line was met in first half of March 201 (50).U CL , Upper control limit; LCL lower control limit.
